# RBPvsMIR: A Computational Pipeline to Identify Competing miRNAs and RNA-Binding Protein Pairs Regulating the Shared Transcripts

**DOI:** 10.3390/genes9090426

**Published:** 2018-08-22

**Authors:** Xing Zhao, Danze Chen, Yujie Cai, Fan Zhang, Jianzhen Xu

**Affiliations:** Computational Systems Biology Lab, Department of Bioinformatics, Shantou University Medical College (SUMC), Shantou 515041, China; zhaoxing0323@163.com (X.Z.); d_z_chen@stu.edu.cn (D.C.); musejie163@163.com (Y.C.); lemon_fan@163.com (F.Z.)

**Keywords:** microRNA, RNA-binding protein, long non-coding RNA, MALAT1

## Abstract

Gene post-transcription regulation involves several critical regulators such as microRNAs (miRNAs) and RNA-binding proteins (RBPs). Accumulated experimental evidences have shown that miRNAs and RBPs can competitively regulate the shared targeting transcripts. Although this establishes a novel post-transcription regulation mechanism, there are currently no computational tools to scan for the possible competing miRNA and RBP pairs. Here, we developed a novel computational pipeline—RBPvsMIR—that enables us to statistically evaluate the competing relationship between miRNAs and RBPs. RBPvsMIR first combines with previously successful miRNAs and RBP motifs discovery applications to search for overlapping or adjacent binding sites along a given RNA sequence. Then a permutation test is performed to select the miRNA and RBP pairs with the significantly enriched binding sites. As an example, we used RBPvsMIR to identify 235 competing RBP-miRNA pairs for long non-coding RNA (lncRNA) MALAT1. Wet lab experiments verified that splicing factor SRSF2 competes with miR-383, miR-502 and miR-101 to regulate MALAT1 in esophageal squamous carcinoma cells. Our study also revealed the global mutual exclusive pattern for miRNAs and RBP to regulate human lncRNAs. In addition, we provided a convenient web server (http://bmc.med.stu.edu.cn/RBPvsMIR), which should accelerate the exploration of competing miRNAs and RBP pairs regulating the shared targeting transcripts.

## 1. Introduction

Gene post-transcription regulation involves several critical regulators such as microRNAs (miRNAs) and RNA-binding proteins (RBPs). miRNAs are short non-coding RNAs that can induce transcript cleavage or protein translation repression by binding to target sites [1]. miRNAs are found to be important in a variety of cellular processes and diseases [2,3]. RNA-binding proteins regulate the synthesis, folding, modification and degradation of RNA molecules throughout the whole lifecycle. The interactions between transcripts and RBPs play a fundamental role in RNA splicing, localization, stability and translation [4,5].

In recent years, accumulated evidences have shown that miRNAs and RBPs can jointly regulate the expression of shared targeting transcripts. For example, Tominaga et al. showed that nucleolin expression is modulated by both the translation-promoting protein HuR and the decay-promoting miR-494. Importantly, they found that miR-494 and RBP HuR functionally competed for the modulation of nucleolin [6]. Interestingly, another group of investigators also found extensive competition between miR-129 and RNA-binding protein HuD. Both miR-129 and HuD can bind the voltage-gated potassium channel Kv1.1 mRNA. When mTORC1 is active, miR-129 inhibits Kv1.1 translation and when mTORC1 is inactive, HuD binds to and promotes Kv1.1 translation. Thus, the expression of Kv1.1 is controlled by miR-129 and HuD in a mTORC1 kinase-dependent manner in neuronal processes [7]. Except for protein-coding mRNAs, other types of transcripts such as long non-coding RNA (lncRNAs) can also be regulated simultaneously by both miRNAs and RBPs [8]. Although this competing relationship between miRNAs and RBPs establishes a novel post-transcription regulation mechanism and individual cases about competing for binding sites between RBPs and miRNAs are continuously reported, there is currently no systematic evaluation for a given transcript. This is mainly hampered by the fact that no computational framework or useful bioinformatics tools for this task [9,10].

Finding sequence motifs plays a central role in both miRNA and RBP binding sites prediction. The first 2–8 bases of a particular mature miRNA sequence, usually referred to as the seed region, is commonly used in almost all bioinformatics algorithms to search for potential miRNA targets [1,11]. Several applications, such as TargetScan (http://www.targetscan.org/) and PITA (https://genie.weizmann.ac.il/pubs/mir07/), are commonly used for miRNA target sites prediction. TargetScan hunts for the presence of conserved 8-mer and 7-mer motifs that match the seed region of each miRNA. Based on 14 sequence features such as site type, predicted seed-pairing stability, local AU content and 3′-UTR target-site abundance, it builds a quantitative model to predict the most effectively targeted mRNAs [12]. In contrast, PITA combines a traditional seed-finding algorithm with a target accessibility thermo-dynamic model to predict the target sites [13]. On the other hand, binding preferences for sequence-specific RBPs are incorporated in most RBP binding site algorithms [14,15]. For example, RBPmap considers the clustering propensity of the binding sites and the overall tendency of regulatory regions to be conserved. It incorporates 94 RNA motifs recognized by mammalian RBPs based on a Weighted-Rank approach [15]. In this study, we developed a sequence motif-based computational pipeline named RBPvsMIR, which enables us to statistically evaluate the competitive relationship between miRNAs and RBPs. Given any RNA molecules, RBPvsMIR first combines with previously successful applications, TargetScan, PITA and RBPmap, to scan the miRNAs and RBP sequence motifs along the query transcripts [12,13,15]. Then RBPvsMIR identifies the adjacent motifs and generates a population of random RNA sequences to evaluate the significance. RBPvsMIR selects the miRNA and RBP pairs that have significantly enriched adjacent sites. We adopted this procedure to analyze all known human functional lncRNAs, with a special focus on one oncogenic lncRNA, MALAT1.

## 2. Materials and Methods

### 2.1. RBPvsMIR Prediction Pipeline

For an arbitrary RNA sequence (in FASTA format), the RBPvsMIR pipeline processes it in two stages. (I) During the scanning stage, the miRNAs binding sites were searched by PITA and/or TargetScan. Users can define the different searching modes, either by individual programs or by combinations/intersection of these programs. RBPmap was used to find the RBP binding sites. ΔΔG is an energetic cutoff score, which is originally used by PITA to evaluate stability between the closed and open forms of miRNA-target duplex [13]. Correspondingly, RBPmap adopts the *p*-value for the result cutoff [15]. RBPvsMIR retains these two parameters for users to test different stringent levels according to their research aims and criteria. Users are also free to choose the distance between the RBP binding sites and miRNA binding sites, either completely overlapped (i.e., distance = 0 bp) or very close sites (i.e., distance < 100 bp). RBPvsMIR also provides rich scanning combinations for RBPs and miRNAs pairs since 677 miRNAs and 94 RBP are incorporated in the pipeline. After scanning motifs, RBPvsMIR then examines the predictions from PITA/TargetScan and RBPmap to record the number of overlapping or adjacent sites (denoted by *O*). (II) During the evaluation stage, a permutation test was performed. *N* simulated query sequences of the same size were also generated by randomly shuffling nucleotides of query sequence, then the same procedure in the scanning stage was applied. The corresponding number of overlapping or adjacent sites was recorded for each simulated sequence (denoted by C1, C2, …, Cn). Accordingly, by comparing the set (C1, C2, …, Cn) and the value *O*, a significance *p*-value is calculated as:

$$\;p=\frac{K}{N}\;$$

where *K* is the number of simulated sequences that is equal to or larger than the number of overlapping or adjacent sites in the query sequence, among all the simulated C1, C2, …, Cn. In this study, *N* is set to 5000. Based on such permutations, it was used to assess whether the overlapping or adjacent sites were significantly enriched. Multiple statistical tests were controlled by the false discovery rate (FDR) [16].

### 2.2. Construction of RBPvsMIR Web Server

The RBPvsMIR web server was implemented using HTML and PHP language and the interface component consists of the web pages designed and implemented in HTML/CSS. Currently, RBPvsMIR focuses on the mammalian genome; therefore, users of RBPvsMIR need to specify the input RNA sequence species (human or mouse), then RBPvsMIR can predict species-specific regulations. MALAT1 and all 135 human functional lncRNAs sequences were downloaded from the lncRNAdb database [17]. All computations were conducted in R and Perl in a Linux environment. The server was tested in Google Chrome, Safari, Mozilla Firefox and Internet Explorer web browsers.

### 2.3. Experimental Confirmation

Human esophageal squamous cell carcinomas cell line TE1 was purchased from the Shanghai Cell Bank, Chinese Academy of Sciences (CAS). Cells were cultured in Roswell Park Memorial Institute (RPMI) 1640 medium (HYCLONE, Logan, UT, USA) with 10% fetal bovine serum. The miRNA primers, synthesized miRNAs mimics, miRNAs inhibitors and negative control were all purchased from Ribobio (Guangzhou, China). Small interfering RNA (siRNAs) were designed and synthesized by GenePharma (Shanghai, China) and the sequences are listed in Table S1. The primers for MALAT1 and SRSF2 are from a previous report and are available in Table S1 [18]. First, 1 × 10^6^ TE1 cells were plated in each well of a six-well plate 24 h prior to transfection. When the cells reached 60–80% confluent, the miRNAs mimics, miRNAs inhibitors and siRNAs were transfected to the indicated final concentration using Lipofectamine RNAiMAX (Thermo Fisher Scientific, Waltham, MA, USA) according to the manufacturer’s recommendations. For quantitative real-time PCR assays, RNA isolation and quantitative real-time PCR (RT-qPCR) were conducted as previous reported [2].

## 3. Results

### 3.1. RBPvsMIR Pipeline and Web Server

**Figure 1:**
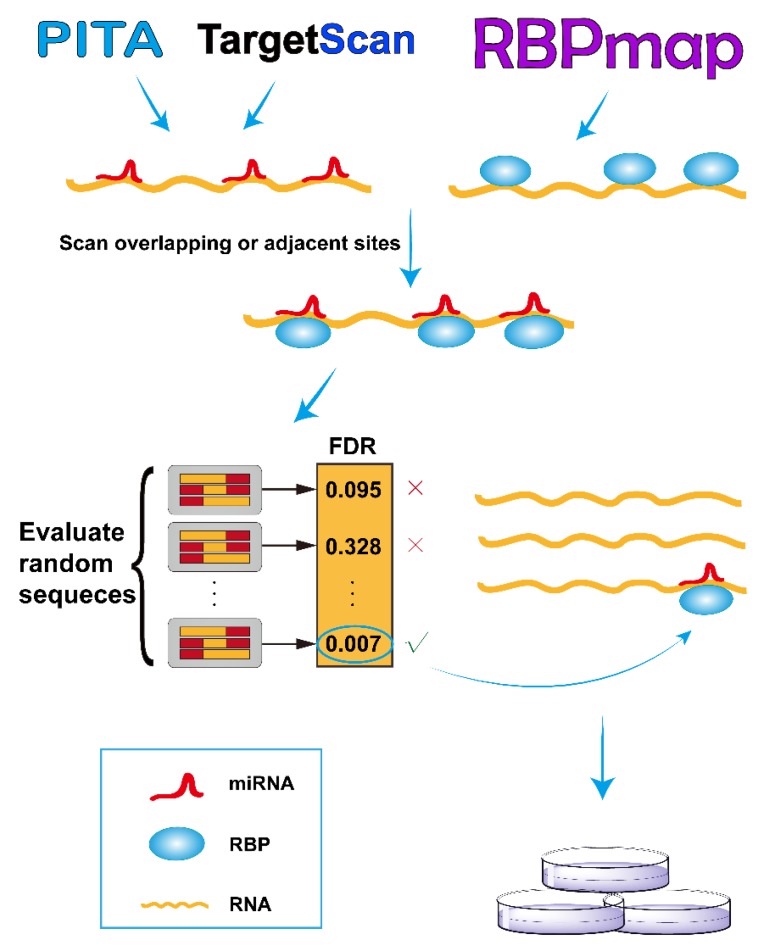
Overview of RBPvsMIR analytical scheme. In the scanning stage, RBPvsMIR initially uses PITA/TargetScan and RBPmap algorithms to respectively search for microRNA (miRNA) and ribosome binding pairs (RBP). Then, during evaluating stage, RBPvsMIR reports the miRNA and RBP pairs with significantly enriched overlapping or very close sites based on a permutation test. Finally, candidate miRNA and RBP pairs are verified by wet experiments. FDR: false discovery rate.

As illustrated in Figure 1, we developed the RBPvsMIR pipeline to hunt for competing miRNA and RBP pairs. RBPvsMIR first uses PITA/TargetScan and RBPmap algorithms to respectively scan for miRNA and RBP motifs along any query RNA sequence. Both of above methods have been widely used in miRNAs and RBP binding sites prediction. They have been tested with RNA-binding experiments and the predictions have been proved to be highly accurate. We reasoned that if the functional sites are competed for by both miRNAs and RBPs, their physical interaction with mRNAs (possibly via steric hindrance) must be close. Based on this concept, RBPvsMIR was designed to find the overlapping or very close miRNA and RBP binding sites (i.e., distance < 100 bp). RBPvsMIR generates 5000 simulated query sequences by shuffling the query sequence. The same searching procedure is applied to the simulated sequences and the corresponding numbers of overlapping or adjacent sites were recorded for each simulated sequence. The fraction of 5000 random sampling sequences that are equal to or larger than the observed number of overlapping or adjacent sites was reported as the significance value. Then, RBPvsMIR selects the miRNA and RBP pairs with significantly enriched overlapping or very close sites (Figure 1).

RBPvsMIR also provides an easy-to-use web server interface (http://bmc.med.stu.edu.cn/RBPvsMIR/). Users first submit query RNA sequences (in FASTA format) as input and select the species and distance options. Users are also free to evaluate their predictions with different computational parameters such as ΔΔG and *p*-value, which are used for thresholding in PITA and RBPmap applications, respectively (see online documentation). When the predictions are finished, RBPvsMIR generates result files for each query sequence which contain: (1) a table including the significant pairs of RBP and miRNAs selected by the user defined cutoff value; (2) a table including each overlapping or adjacent functional site between each pair of RBP and miRNA, together with all of the resultant variables (ΔΔG, *p*-value, real number of overlapping or adjacent sites and observed number of sequences that are equal to or larger than the real one in the simulated data and FDR value); and (3) an interaction network, which describes the mutual relationships among all the significant RBPs and miRNAs (see below). The server automatically sends the emails to users when all of the results are ready.

### 3.2. Predicting and Confirming the Competing RNA-Binding Protein and microRNA Pairs on Long Non-coding RNA MALAT1

**Figure 2:**
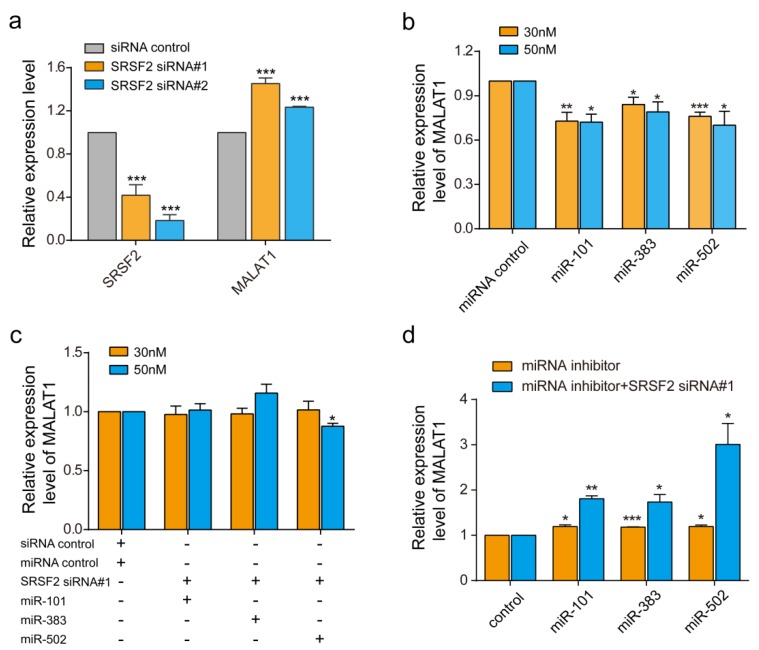
Experimental confirmation of predicted competition between miRNAs and RBPs. (**a**) SRSF2 and MALAT1 expression in TE1 cells treated with two small interfering RNA (siRNA)-SRSF2 and control siRNAs. (**b**) Quantitative real time polymerase chain reaction (qRT-qPCR) for MALAT1 in TE1 cells after transfection with miR-101, miR-383 and miR-502. (**c**) qRT-PCR of MALAT1 in TE1 cells after co-transfection with both siRNA-SRSF2 and miRNAs. (**d**) qRT-PCR of MALAT1 in TE1 cells after co-transfection with both siRNA-SRSF2 and miRNAs inhibitors. * *p* < 0.05, ** *p* < 0.01, *** *p* < 0.001. The concentrations of miRNA mimics were tested at both 30 nM and 50 nM. The concentrations of miRNA inhibitors were tested at 100 nM. Data are the means ± standard error of mean (s.e.m.) of three experiments.

Metastasis associated lung adenocarcinoma transcript 1, MALAT1, is a relatively large lncRNA with more than 8000 bp. We used it as an example to assess RBPvsMIR performance. We used a stringent cutoff with *p* ≤ 0.01, ddg ≤ −6, distance = 0 bp and FDR ≤ 0.05. In total, RBPvsMIR found 866 RBP and miRNA overlapping binding sites, which accounted for 235 RBP and miRNAs pairs (File S1). The mutual interactions involved 61 unique RBPs and 88 unique miRNAs. Among all of the RBPs which potentially interacted with MALAT1, we were particularly interested in the serine/arginine (SR) splicing factors 2, SRSF2, which is a member of the splicing factors. SRSF2 is known to play an essential role in constitutive and alternative pre-mRNA splicing and has been linked to diseases [18,19]. Previously, it was proved that MALAT1 interacts with SR splicing factors including SRSF2 to regulate tissue- or cell-type-specific alternative splicing in a concentration- and phosphorylation-dependent manner [20]. Consistent with previous reports, we found that inhibiting SRSF2 with two siRNAs significantly induced the expression of MALAT1 about 1.45-fold (siRNA#1) and 1.2-fold (siRNA#2), respectively (Figure 2a). Interestingly, SRSF2 could compete with several miRNAs in our prediction results (FDR ≤ 0.05) (File S1). We randomly selected two miRNAs, miR-383 and miR-502, as test examples for verification. In addition, we also tested the miR-101-SRSF2 competing relationship because miR-101 was found to inhibit MALAT1 in previous reports and with a low FDR in our prediction (FDR = 0.19) [21]. After miRNA mimics transfections, the miRNAs expression increased to a 12-fold (miR-101) or a 100-fold (miR-383 and miR-502) (Figure S1a). We then evaluated the individual regulation of miRNAs on the expression of MALAT1 by quantitative real-time RT-PCR. As can be seen from Figure 2b, compared with the vehicle control, there are significant decreases in MALAT1 mRNA levels in cells transfected with these miRNAs (Figure 2b). These results suggested that miRNAs generally down-regulate MALAT1, perhaps due to the induction of RNA degradation. Since inhibiting SRSF2 could induce the expression of MALAT, we then co-transfected both siRNA-SRSF2 and miRNAs to see if there are antagonistic effects among the SRSF2 and the three assessed miRNAs. Indeed, although individual miRNA reduced the MALAT1 level by ~25%, the co-transfection of SRSF2 siRNA with miRNAs reversed the inhibition of MALAT1 (Figure 2b,c). To further confirm above results, we then used miRNA inhibitors to test effects of the endogenous pool of miRNAs. The transfections of miRNA inhibitors significantly reduced the endogenous expression of miR-101, miR-383 and miR-502 (Figure S1b). Meanwhile, the expression of MALAT1 was increased to ~1.2 folds after miRNA inhibitions (Figure 2d). When both siRNA-SRSF2 and inhibitors were co-transfected, consequently the expression of MALAT1 was increased further to ~1.7 folds (miR-101 and miR-383) or 2.8 folds (miR-502). Thus, both miRNA over-expression and endogenous miRNAs inhibition demonstrated that miR-383, miR-101 and miR-502 compete with SRSF2 to regulate MALAT1.

### 3.3. A Computational Landscape of Competing microRNA and RNA-Binding Protein Pairs on Human Functional Long Non-coding RNAs

**Figure 3:**
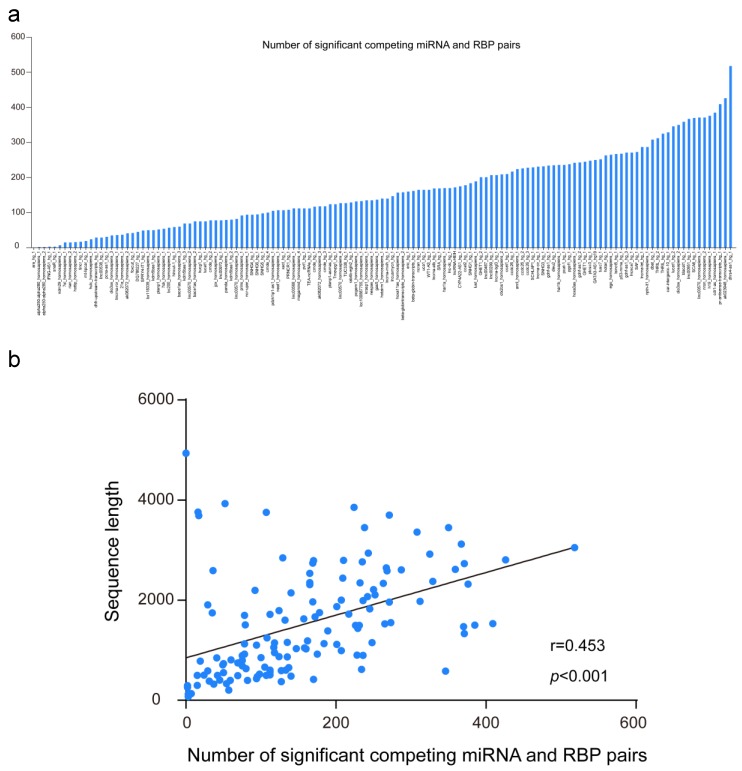

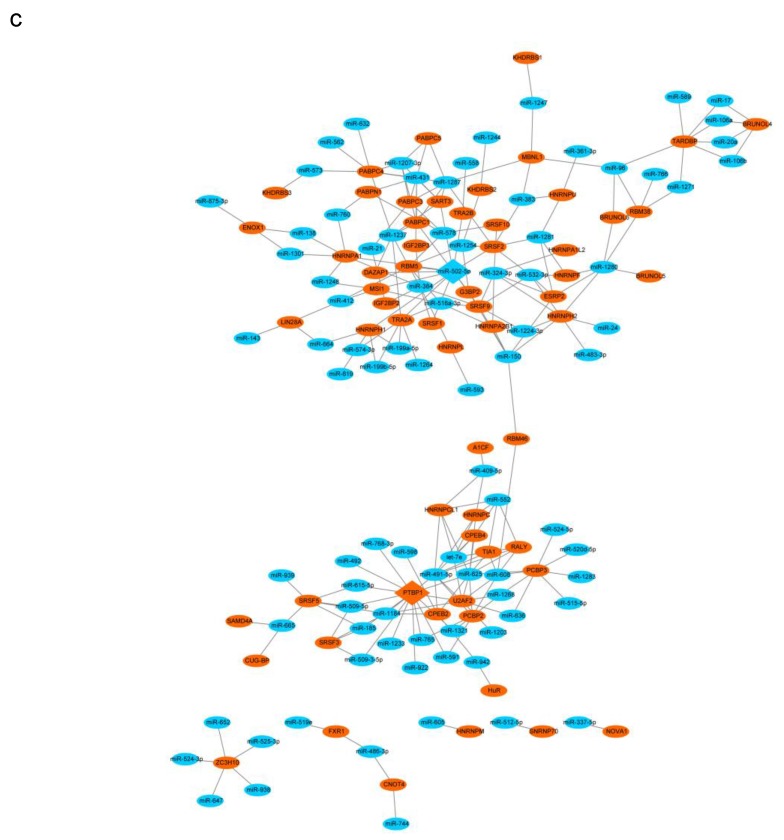
Global patterns of the competing miRNA and RBP pairs on human functional long non-coding RNA (lncRNAs). (**a**) The range of numbers of significant competing miRNA and RBP pairs for human functional lncRNAs. (**b**) the number of competing miRNA and RBP pairs is correlated with the sequence length of lncRNAs. (**c**) the interacting network of competing miRNAs and RBPs along MALAT1 sequence. Orange and blue circular nodes represent miRNAs and RBPs, respectively. Edges represent the competing relationships among them. Note that diamond nodes are the most connected miRNAs and RBPs in the network.

Finally, we downloaded known human functional lncRNAs sequences from the lncRNAdb database. The average sequence length of the 135 lncRNAs is ~1.5 Kb RBPvsMIR was applied to predict the competing miRNA and RBP pair for them. As illustrated in Figure 3a, the range of the number of significant pairs is much wider, from a minimum value of zero to a maximum greater than 500 (with an average of 162 pairs for each human lncRNA). As expected, the number of competing pairs is significantly correlated with the sequence length of lncRNAs (Figure 3b, Pearson correlation r = 0.453, *p* < 0.001). On the other hand, although 13,887 unique competing miRNA and RBP pairs were found for all functional lncRNAs, only ~3.3% of them are shared by five or more lncRNAs (452 out of 13,887 pairs). Among them, poly(rC) binding protein PCBP2 and hsa-miR-1207-5p make up the most mutual exclusive pair, which may competitively regulate 18 lncRNAs (File S2).

Representing RBPs and miRNAs as nodes and their competing relationships as edges, we focused on the general pattern of competing miRNA and RBP pairs on MALAT1. As demonstrated in Figure 3c, nearly 90% of these RBPs and miRNAs are interconnected into a global network, which suggests that these miRNAs and RBPs are intensively involved in MALAT1 post-transcription regulation. From the global network for MALAT1 regulation, we also found that there are a few nodes such as miR-502 that compete with multiple RBPs. Alternatively, there are also RBPs nodes such as PTPB1 that have antagonistic effects on multiple miRNAs. These results illustrated the complex mutual exclusive rules among RBPs and miRNAs for MALAT1 regulation.

## 4. Discussion

In theory, both miRNAs and RBPs rely on sequence motifs to recognize their functional targets, suggesting that, in some cases, RBPs and miRNAs could compete with each other because of steric hindrance. In spite of the drastic expansion of public software for the individual prediction of miRNAs and RBPs, there are few platforms that analyze them simultaneously. We sought to address these limitations by developing a novel, sequence-based pipeline, RBPvsMIR, to detect overlapping or very close binding sites and find competing miRNA and RBP pairs.

As an example, we used RBPvsMIR to identify competing RBP and miRNA pairs on lncRNA MALAT1. MALAT1 is an established oncogenic lncRNA, which plays an important role in many cancers [22]. In esophageal squamous cell carcinomas, MALAT1 functions as a competing endogenous RNA (ceRNA) to sequester miRNAs [21]. In colorectal cancer, MALAT1 could also bind to splicing factor proline and glutamine rich (SFPQ), thus increasing cell proliferation and migration [23]. Although several miRNAs and RBPs have been linked with this lncRNA, there is no systemic evaluation of their antagonistic mechanism. With the help of RBPvsMIR, we totally found 235 RBP and miRNA competing pairs. We also used RT-qPCR and siRNA transfection assays to verify that splicing factor SRSF2 competes with miR-101, miR-383 and miR-502 on MALAT1. This finding provided clues for a novel molecular mechanism governing the post-transcription regulation of MALAT1 in esophageal cancer cells. Furthermore, extending the pipeline to all known human lncRNAs revealed more extensive competition among RBPs and miRNAs than previously thought and shed light on the landscape of miRNA and RBP mutual exclusive regulation on lncRNAs.

RBPvsMIR provides a user-friendly interface for applying this search algorithm to any RNA sequence. To the best of our knowledge, there is only one database, named DosiR, that has some related functionality. Users can extract RBPs and miRNAs binding sites from available human crosslinking and immunoprecipitation (CLIP) data in DosiR [24]. However, the available RBP regulators are limited in DosiR. In addition, there is no statistical evaluation of the sites. On the contrary, RBPvsMIR is a sequence-based prediction tool and can be used for any transcripts. Besides, RBPvsMIR uses a sampling procedure to assess the statistical significance of overlapping sites among RBPs and miRNAs. In fact, only scanning the overlapping or very close binding sites without considering the statistical significance will produce thousands of predictions in the MALAT1 examples. RBPvsMIR has eliminated over 90% of non-significant sites, many of which would be false positives. Currently, RBPvsMIR focuses on human and mouse genomes, as mammalian RBP motifs have been widely reported. When other species’ RNA motifs are accumulated, RBPvsMIR can be tailored to further predict species-specific regulation. Another update in the future includes the incorporation of expression profiles of miRNAs and RBPs when stoichiometry is considered.

It has been clearly demonstrated that miRNAs are bound by one special type of RNA-binding protein, argonaute and work as a guide for argonaute to identify complementary target mRNAs [1,25]. Therefore, in essence, the competition for binding between RBPs and miRNAs can be viewed as one specialized competition among RBPs. Our pipeline can also be extended to predict the mutual exclusive pattern between RBPs, leading to novel insights into gene regulatory networks.

## Supplementary Materials

The following are available online at link http://www.mdpi.com/2073-4425/9/9/426/s1, **Table S1**: siRNA sequences and primers used in this study; **File S1**: Prediction results for lncRNA MALAT1; **File S2**: Prediction results for all functional lncRNAs; **Figure S1**: Supplementary Figures.
