# Supplementary material for: RBPvsMIR: A Computational Pipeline to Identify Competing miRNAs and RNA-Binding Protein Pairs Regulating the Shared Transcripts
Source: Genes (Basel). 2018 Aug 22;9(9):426. doi: 10.3390/genes9090426 (PMC6162414; doi:10.3390/genes9090426)
Supplement: Supplementary file 1 [file genes-09-00426-s001.zip › genes-337412 - Supplementary - after proofs/Figure S1.docx]

Supplementary Figure S1

The miRNAs expressions were measured by qRT-PCR after miRNA mimics or inhibitor transfections in TE1 cells


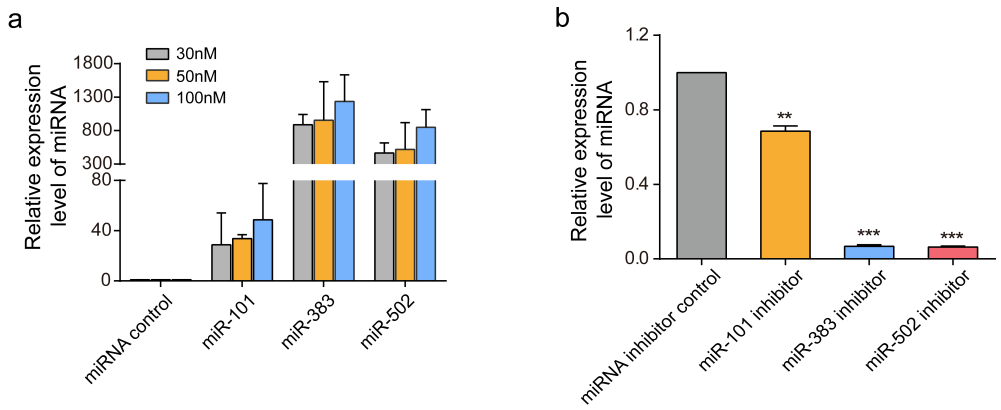


(**a**) qRT-PCR of miRNAs expression after miRNA mimics transfections; (**b**) qRT-PCR of miRNAs expression after miRNA inhibitors transfections;
