# Supplementary material for: RBPvsMIR: A Computational Pipeline to Identify Competing miRNAs and RNA-Binding Protein Pairs Regulating the Shared Transcripts
Source: Genes (Basel). 2018 Aug 22;9(9):426. doi: 10.3390/genes9090426 (PMC6162414; doi:10.3390/genes9090426)
Supplement: Supplementary file 1 [file genes-09-00426-s001.zip › genes-337412 - Supplementary - after proofs/Table S1.docx]

**Supplementary Table S1. Sequences(5'->3') used in this study**

**siRNA**

|  |  |
| --- | --- |
| SRSF2 siRNA#1  SRSF2 siRNA#2  Control siRNA | GAGGACGCUAUGGAUGCCAUGGACG  UCGACCGAGAUCGAGAACGAG  UUCUCCGAACGUGUCACGUTT |

**Primer pairs for qRT-PCR**

| SRSF2-F | CCACTCAGAGCTATGAGCTACG |
| --- | --- |
| SRSF2-R | ACTCCTTGGTGTAGCGATCC |
| MALAT1-F | GACGGAGGTTGAGATGAAGC |
| MALAT1-R | ATTCGGGGCTCTGTAGTCCT |
| human-ACTB-F | CATGTACGTTGCTATCCAGGC |
| human-ACTB-R | CTCCTTAATGTCACGCACGAT |
